# Supplementary material for: Promotive Role of 5-Aminolevulinic Acid or Salicylic Acid Combined with Citric Acid on Sunflower Growth by Regulating Manganese Absorption
Source: Antioxidants (Basel). 2023 Feb 25;12(3):580. doi: 10.3390/antiox12030580 (PMC10045730; doi:10.3390/antiox12030580)
Supplement: Supplementary file 1 [file antioxidants-12-00580-s001.zip › Supplementary Materials.pdf]

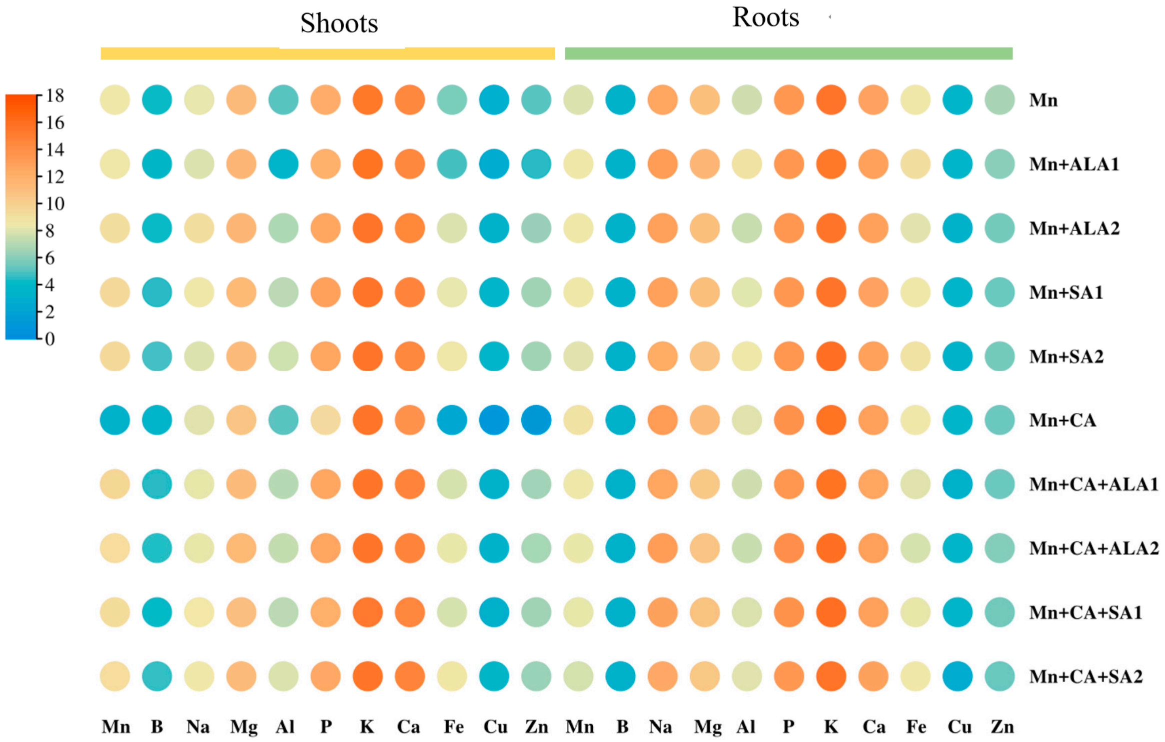

Figure S1. The heatmap of effects of 5-aminolevulinic acid or salicylic acid (ALA/SA) combined with citric acid (CA) on manganese (Mn) and other elements concentration on shoots and roots of Mn treated sunflower plants. Mn means that plants exposed to 0.8 g kg^{-1} of Mn for 7 days; Mn + ALA1/ALA2 indicates spraying with 10/20 mg L^{-1} ALA after Mn treatment for 7 days; Mn + SA1/SA2 indicates spraying with 50/100 mg L^{-1} SA after Mn treatment for 7 days. Mn + CA indicates plants treated with 20 mmol kg^{-1} CA 7 days after Mn treatment. Mn + CA + ALA/SA treatment indicates plants treated with ALA/SA for one week after CA treatment. Data are the means of three replicates by Log_2 fold change.

(a)

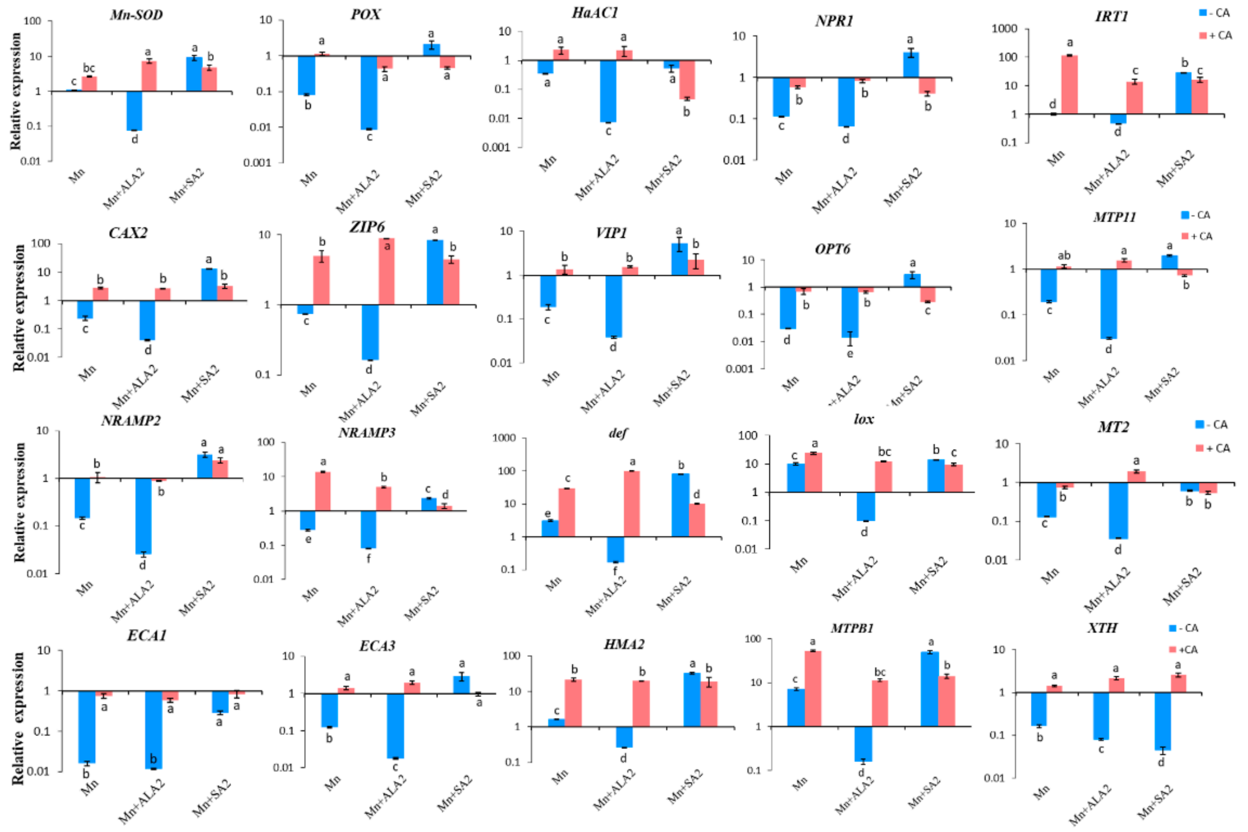

(b)

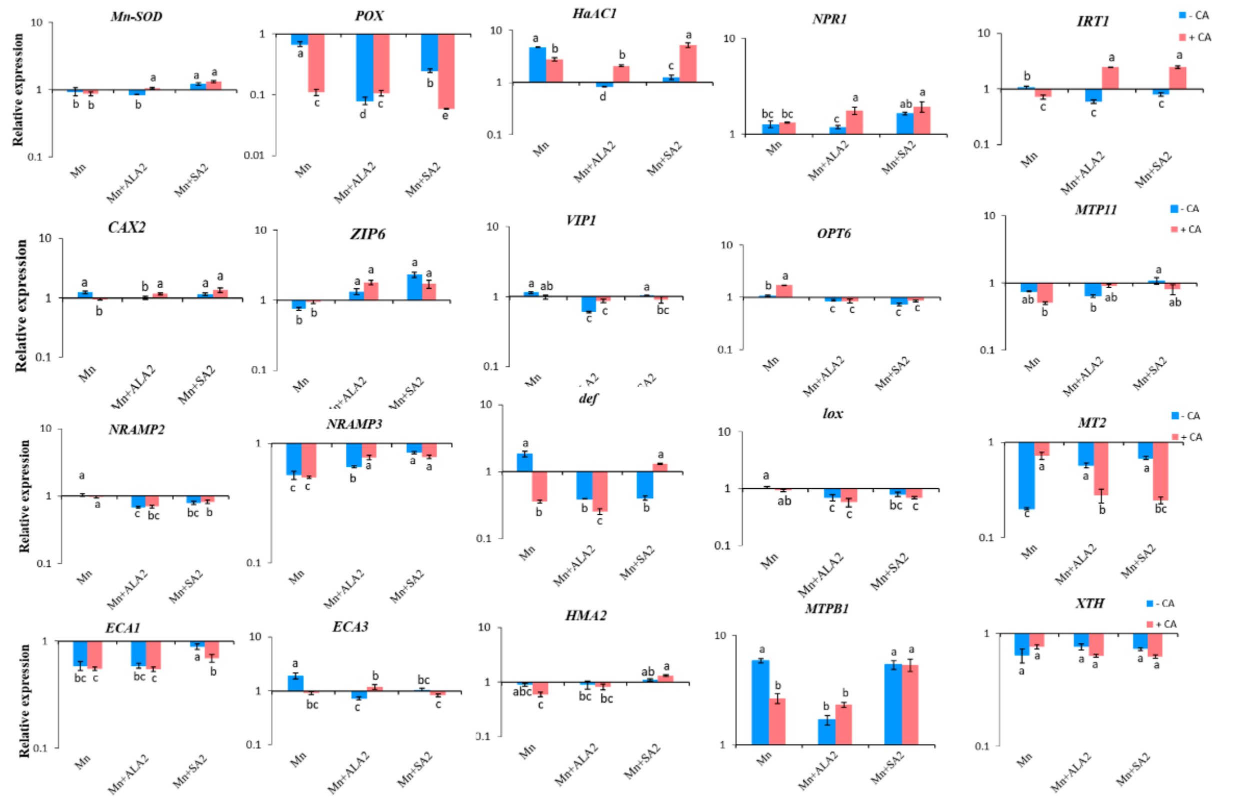

Figure S2. Effects of 5-aminolevulinic acid or salicylic acid (ALA/SA) combined with citric acid (CA) on tolerant-related gene expression patterns in the leaves (a) and roots (b) of manganese (Mn)-treated sunflower plants. CA, 20 mmol kg^{-1} citric acid treatment. CK, control plants without any treatment; Mn, plants exposed to 0.8 g kg^{-1} of Mn for 7 days; Mn + CA, plants

treated with CA 7 days after Mn treatment; Mn + CA + ALA2, plants treated with 20 mg L^{-1} ALA for one week after CA treatment; Mn + CA + SA2, plants treated with 100 mg L^{-1} SA for one week after CA treatment. The relative expression levels are calculated by $2^{-\Delta\Delta Ct}$ method compared with that of *EF-1α* with three replicates. The results are represented by means ± standard errors. The expression level in control is normalized as "1". Means are denoted by the same letter when there is no significant difference at $P < 0.05$ as determined by Duncan's multiple range test.

Table S1 The combinations of different treatments in the experiment.

Agentia / Treatments	CK	Mn (800 mg kg^{-1})	CA (20 mmol kg^{-1})	ALA1 (10 mg L^{-1})	ALA2 (20 mg L^{-1})	SA1 (50 mg L^{-1})	SA2 (100 mg L^{-1})
1	√						
2				√			
3					√		
4						√	
5							√
6		√					
7		√		√			
8		√			√		
9		√				√	
10		√					√
11		√	√				
12		√	√	√			
13		√	√		√		
14		√	√			√	
15		√	√				√

Table S2 Primers of sunflower genes used for the RT-qPCR analysis

Gene	Description	PCR primers	Accession No.
EF-1α	elongation factor 1 alpha（reference gene）	GGATACAACCCCGACAAA<br>CCTGAAGTGGGAGACGGA	LOC110904321
Mn-SOD	superoxide dismutase [Mn], mitochondrial	TGAATGCTGAAGGTGCTG<br>CCCAAACATCTATGCCAAT	LOC110872653
POX	basic peroxidase	CAGCTCGTGATGCTTCTGTC<br>GCGACCATTTCTCTCGTGTT	LOC110921228
HaAC1	auxin-induced protein PCNT115	AGTGTCCATCCAATAACTGCCA<br>ACCTTGGAAAGTTCGGACGAT	LOC110908643
NPR1	BTB/POZ domain and ankyrin repeat-containing protein NPR1	TGGGTATGATGCGTTTCTCGT<br>AGTTGAGAAGACGACGCTGA	LOC110898811
CAX2	vacuolar cation/proton exchanger 3	CCCACTTCAACCCACCAATC<br>GGTGGGCAGATCATACTGGA	LOC110935436
IRT1	probable zinc transporter 10	ACTCATGGTGGACTCGATGG<br>TGGCTACGACACGATAACGA	LOC110872294
OPT6	oligopeptide transporter 6	AAGACGAAAGGTGCATTCGG<br>TAGCAACACCACAAGCCAAC	LOC110936446
NRAMP2	metal transporter Nramp2	CTGATGTGGGCGACGGTAAT<br>AGCAATCGCACTCCCAATCA	LOC110915178
NRAMP3	metal transporter Nramp1-like	CGAGTACGGAACCAATACGC<br>GCTGAACAAGCAAGCCGATA	LOC110903996
ZIP6	zinc transporter 6, chloroplastic	CTTCACAAGCGTCATCGGAA<br>GAGATGACACCTGGCAATCG	LOC110869063
VIP1	transcription factor VIP1	ACCATGCTCCAGCGGGATA<br>AACATATGCGCATGCTGAGG	LOC110942245
MTPB1	metal tolerance protein B	GTCTTGTGACCGTTGTTGCT<br>AAATGGGCTGCATCCGTAAG	LOC110940073
MTP11	metal tolerance protein 11	GATGGATCCCGTTGGAGCTA<br>GCCCGTACGGTATCAATGTG	LOC110877757
def	defensin (anther-specific protein SF18-like)	TCCGGAACATGTGGCAAGAC<br>CTTCTCCTTGGCGAGCTCTT	LOC110893837
lox	lipoxygenase 2, chloroplastic	GTGTCATCACCATCCAAC<br>GCATAAGCCTTCACTGTCT	LOC110898679
ECA1	calcium-transporting ATPase 1, endoplasmic reticulum-type	GGCTGGCCAAGAAACTTCAA<br>TACACCCGAGAGTCTCAACG	LOC110908087
ECA3	calcium-transporting ATPase 3, endoplasmic reticulum-type	GGCTGGCCAAGAAACTTCAA<br>TACACCCGAGAGTCTCAACG	LOC110894822
HMA2	cadmium/zinc-transporting ATPase HMA2	TCAAATGGTTGGCACTCGGA<br>TCAGTGCTTCATAGCAGGCTT	LOC110929174
MT2/mt	metallothionein-like protein type 2	CTTGCTGCAGCGGAAAGT<br>GTTTCCACCCTCAGCAACAA	LOC110873772
XTH	xyloglucan endotransglucosylase/hydrolase protein 9	GTGTTGGTAACAGGGAGCAG<br>TACACCCATGGCTTGGTCTT	LOC110911614

Table S3 Pearson correlation coefficients among plant biomass (DW), POD in root, oxidative damage in root, Mn uptake, Mn translocation in sunflower under Mn treatment (n=3).

	Shoot biomass	Root biomass	POD in leaves	APX in leaves	MDA in leaves	H_2O_2 in leaves	Shoot Mn concentrations	Root Mn concentrations	TF	RE
Shoot biomass	1									
Root biomass	0.497**	1								
POD in leaves	0.039	0.092	1							
APX in leaves	0.453*	-0.026	-0.490**	1						
MDA in leaves	0.233	-0.277	-0.330	0.410*	1					
H_2O_2 in leaves	-0.549**	-0.605**	-0.206	-0.164	0.457*	1				
Shoot Mn concentrations	-0.730**	-0.383*	0.444*	-0.620**	-0.276	0.368*	1			
Root Mn concentrations	0.705**	0.620**	0.084	0.381*	-0.056	-0.598**	-0.407*	1		
Translocation factor	-0.855**	-0.577**	0.260	-0.648**	-0.146	0.586**	0.879**	-0.717**	1	
Remove efficiency	-0.509**	-0.207	0.540**	-0.562**	-0.235	0.175	0.945**	-0.194	0.718**	1

* and ** represent significant correlations at level $p < 0.05$ and $p < 0.01$, respectively.

Table S4 Pearson correlation coefficients among different metal elements, including Mn, B, Na, Mg, Al, P, K, Ca, Fe, Cu and Zn (n=3) in sunflower shoot.

	Mn concentration	B	Na	Mg	Al	P	K	Ca	Fe	Cu	Zn
Mn	1										
B	0.645^{**}	1									
Na	0.202	0.034	1								
Mg	0.379^{*}	0.491^{**}	0.199	1							
Al	0.656^{**}	0.777^{**}	0.217	0.155	1						
P	0.823^{**}	0.701^{**}	0.248	0.588^{**}	0.584^{**}	1					
K	-0.042	-0.014	-0.059	0.420^{*}	-0.156	0.246	1				
Ca	0.545^{**}	0.611^{**}	0.206	0.794^{**}	0.441^{*}	0.609^{**}	0.041	1			
Fe	0.734^{**}	0.815^{**}	0.211	0.316	0.947^{**}	0.720^{**}	-0.010	0.522^{**}	1		
Cu	0.764^{**}	0.895^{**}	0.175	0.477^{**}	0.810^{**}	0.845^{**}	0.056	0.643^{**}	0.900^{**}	1	
Zn	0.872^{**}	0.720^{**}	0.322	0.314	0.793^{**}	0.797^{**}	-0.130	0.514^{**}	0.850^{**}	0.813^{**}	1

* and ** represent significant correlations at level $p < 0.05$ and $p < 0.01$, respectively.

Table S5 Pearson correlation coefficients among different metal elements, including Mn, B, Na, Mg, Al, P, K, Ca, Fe, Cu and Zn (n=3) in sunflower root.

	Mn concentration	B	Na	Mg	Al	P	K	Ca	Fe	Cu	Zn
Mn	1										
B	0.049	1									
Na	0.358	-0.045	1								
Mg	0.468**	0.058	0.568**	1							
Al	0.192	0.144	0.008	0.398*	1						
P	0.017	0.365*	0.258	0.010	0.099	1					
K	-0.045	0.284	-0.394*	-0.383*	0.037	0.540**	1				
Ca	0.268	0.504**	-0.063	0.361	0.299	0.262	0.418*	1			
Fe	0.161	0.178	-0.202	0.262	0.858**	0.023	0.091	0.281	1		
Cu	0.485**	0.155	0.277	0.523**	0.288	0.244	0.215	0.493**	0.266	1	
Zn	-0.108	0.246	-0.132	0.042	-0.046	-0.082	0.090	0.211	0.160	0.248	1

* and ** represent significant correlations at level $p < 0.05$ and $p < 0.01$, respectively.
